# Effects of training in pairs versus training alone on reaching proficiency in minimally invasive Roux-en-Y-gastric bypass on a virtual reality trainer in medical students: a randomized-controlled trial

**DOI:** 10.1007/s00464-025-11701-9

**Published:** 2025-04-14

**Authors:** Amila Cizmic, Paulina Reichert, Frida Häberle, Anas A. Preukschas, Frank Pianka, Arianeb Mehrabi, Anna Nießen, Beat P. Müller-Stich, Thilo Hackert, Rainer Grotelüschen, Felix Nickel

**Affiliations:** 1 Department of General, Visceral and Thoracic Surgery, University Medical Center Hamburg-Eppendorf, Martinistraße 52, 20251 Hamburg, Germany; 2 Department of General, Visceral and Transplantation Surgery, Heidelberg University Hospital, Heidelberg, Germany; 3 Department of Digestive Surgery, University Digestive Healthcare Center Basel, Basel, Switzerland

**Keywords:** Bariatric surgery, VR training, Simulation MIS training, RYGB

## Abstract

**Background:**

Minimally invasive surgery (MIS) is the standard approach in bariatric surgery. The most common bariatric procedures are sleeve gastrectomy and Roux-en-Y-Gastric Bypass (RYGB). Simulation training, including virtual reality (VR), is useful when learning MIS. Training in pairs has proven beneficial in acquiring basic MIS skills. However, this has not been tested on more complex procedures such as MIS RYGB. The study aimed to assess the learning effects of training MIS RYGB on a VR trainer in pairs compared to solo training.

**Methods:**

Medical students (n = 60) were randomized into the intervention group, trained in pairs (n = 30), and the control group, trained solo (n = 30). Both groups needed to train MIS RYGB on a VR trainer under the supervision of trained tutors until proficiency was reached. The MIS RYGB proficiency was defined as 105/110 points according to the Bariatric Objective Structured Assessment of Technical Skills (BOSATS) score. The primary outcome was the number of exercise repetitions until proficiency was reached. Secondary outcomes compared the BOSATS scores, bleeding incidents, and the validated score on current motivation.

**Results:**

The intervention group achieved proficiency with significantly fewer repetitions than the control group (p = 0.002). Most participants in the intervention group reached proficiency by the fifth repetition, and none required an eighth repetition. The intervention group had better BOSATS scores than the control group after the second, fourth, and fifth MIS RYGB (91.1 ± 6.4 vs. 87.1 ± 7.0 points, p = 0.025; 104.0 ± 4.7 vs. 100.3 ± 6.1 points, p = 0.014; 106.2 ± 2.8 vs. 101.9 ± 5.8 points, p = 0.026), respectively. Additionally, the intervention group experienced fewer bleeding complications in the fifth and sixth MIS RYGB repetitions than the control group (2 vs. 10, p = 0.001; 0 vs. 8, p < 0.001, respectively).

**Conclusions:**

Training MIS RYGB on a VR trainer in pairs enables trainees to reach procedural proficiency with fewer exercise repetitions than training alone.

**Supplementary Information:**

The online version contains supplementary material available at 10.1007/s00464-025-11701-9.

Obesity has become a significant global health concern, with its prevalence rising over the past few decades [1, 2]. In 2022, more than 1 billion people worldwide were living with obesity, equating to one in every eight individuals [3]. Bariatric surgery is a well-established treatment for selected individuals with severe obesity, offering significant and sustained weight loss, along with improvements in obesity-related comorbidities such as type 2 diabetes, hypertension, and sleep apnea [4, 5].

Minimally invasive surgery (MIS) is the standard approach in bariatric surgery [6–8]. The two most common bariatric surgical procedures are sleeve gastrectomy and Roux-en-Y-gastric bypass (RYGB) [9]. To master bariatric surgeries, surgeons need to develop specific skills related to the MIS aspects of the procedures [10–12]. The learning curve definitions for sleeve gastrectomy and RYGB are heterogeneous. However, MIS training curricula and high procedure volume are crucial for successful outcomes during the learning curve in both bariatric procedures [12].

Training curricula in MIS are often compiled from E-learning modules, box trainers, virtual reality (VR) trainers, computer simulators, and cadaver and animal models [13–17]. VR training positively affects MIS training by reducing operation times and teaching practical MIS skills [18–20]. Moreover, previous studies have shown that practicing MIS procedures on a VR trainer benefits the learning curve in bariatric surgery [21–23]. Implementing specific learning tools, such as simulation training and checklists, improves cognitive and practical skills in bariatric surgery [24].

Furthermore, positive effects of training MIS skills in novices in pairs compared to solo training have been reported in the scientific literature [25]. The MIS training in pairs could be used for invaluable mutual feedback from the participants, potentially benefiting the procedure’s learning curve. Procedural assessment should be standardized and objective to allow proficiency in the individual steps of the procedure. In the case of MIS RYGB, that would be the Bariatric Objective Structured Assessment of Technical Skills (BOSATS) score [24]. However, no study has been conducted on the learning effects of training in pairs versus solo in MIS RYGB on a VR trainer in novices.

The primary aim of this study was to investigate whether training MIS RYGB in pairs on a VR trainer is advantageous or whether comparable results can be achieved while training solo.

## Methods

### Study design

**Fig. 1:**
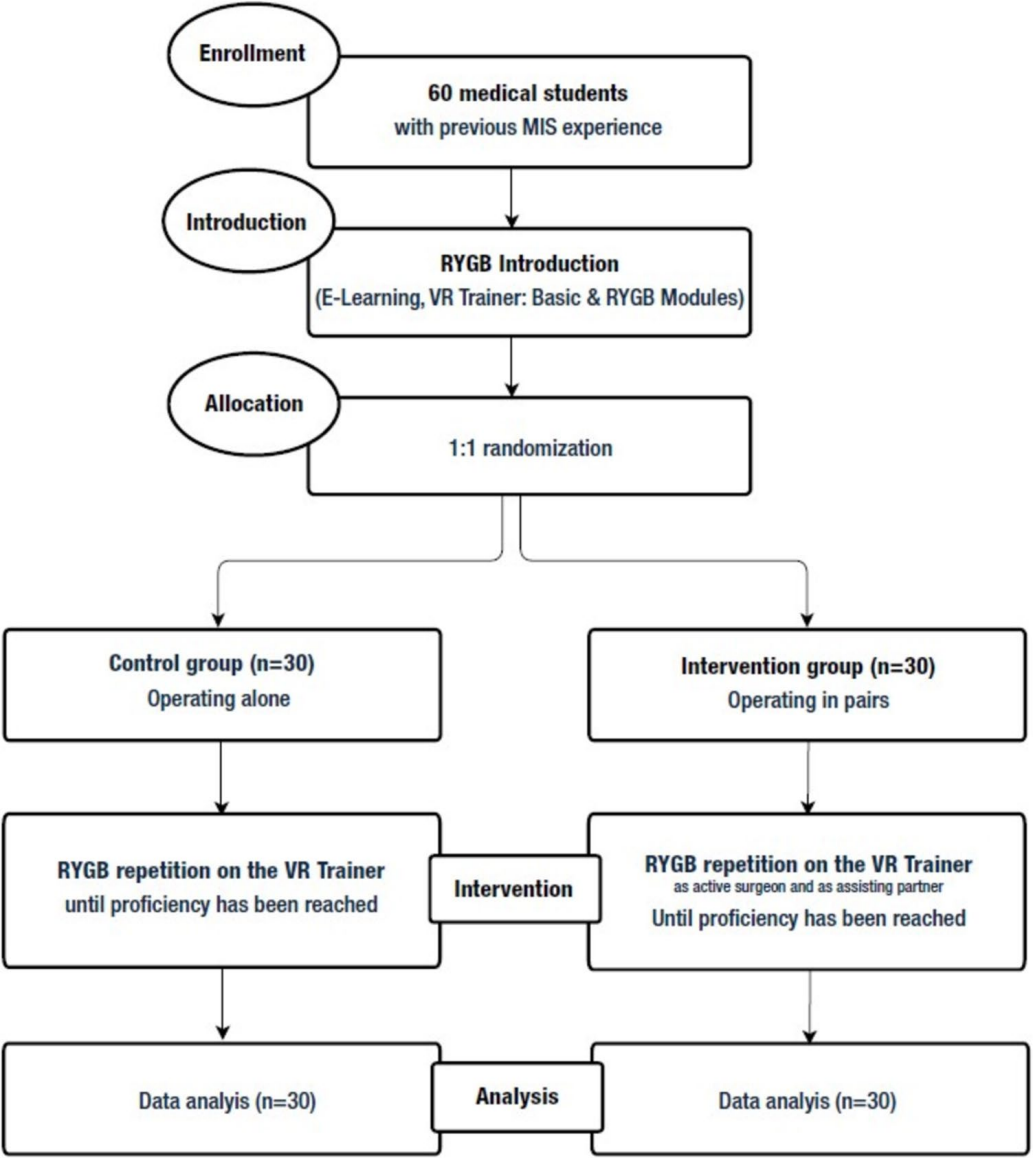
Flow chart of the study

The study was designed as a prospective, single-center, two-arm, randomized-controlled study reported in line with the CONSORT guidelines for randomized-controlled trials (Fig. 1) [26]. It was conducted at a dedicated MIS training center as part of an advanced MIS training curriculum for medical students. The training curriculum was specifically developed for the purpose of the study and was not previously validated by other studies. The local ethics committee approved the conduction of the study (S-436/2018). The study was not registered in a trial registry. Study protocol is available as Supplementary Material 1.

### Inclusion and exclusion criteria

Medical students who had successfully completed an MIS basic training course were included in the study. This was ensured by reviewing their notes and verifying completion, as the basic MIS training course is one of the mandatory courses in the Medical Faculty at the University of Heidelberg. Medical students needed to master basic MIS skills such as camera guidance, PEG transfer, cutting, intracorporeal knotting, and laparoscopic cholecystectomy on the VR Trainer and ex-vivo porcine liver in order to complete the basic MIS training course. The exclusion criteria were unwillingness to participate in the study and lack of previous MIS training.

### Planned sample size

Sample size calculation was based on a previous study performed [27]. Initially, 72 (n = 72) participants were planned to be included in the study. However, the study was performed as a part of an advanced MIS course for medical students, and only 60 (n = 60) students were enrolled.

### Study flow

All enrolled participants acquired theoretical knowledge through a 2-h E-learning session about the MIS RYGB (http://www.websurg.com) under the supervision of trained tutors [28]. The E-learning modules included anatomy studies, illustrations, and videos of the MIS RYGB. An experienced board-certified bariatric surgeon previously trained the tutors in performing and teaching MIS RYGB on a VR trainer.

Afterward, the participants were trained in Basic MIS and four-step MIS RYGB modules on a VR trainer (Simbionix LAP Mentor) under the supervision of trained tutors. Each participant completed both modules once and individually.

**Fig. 2:**
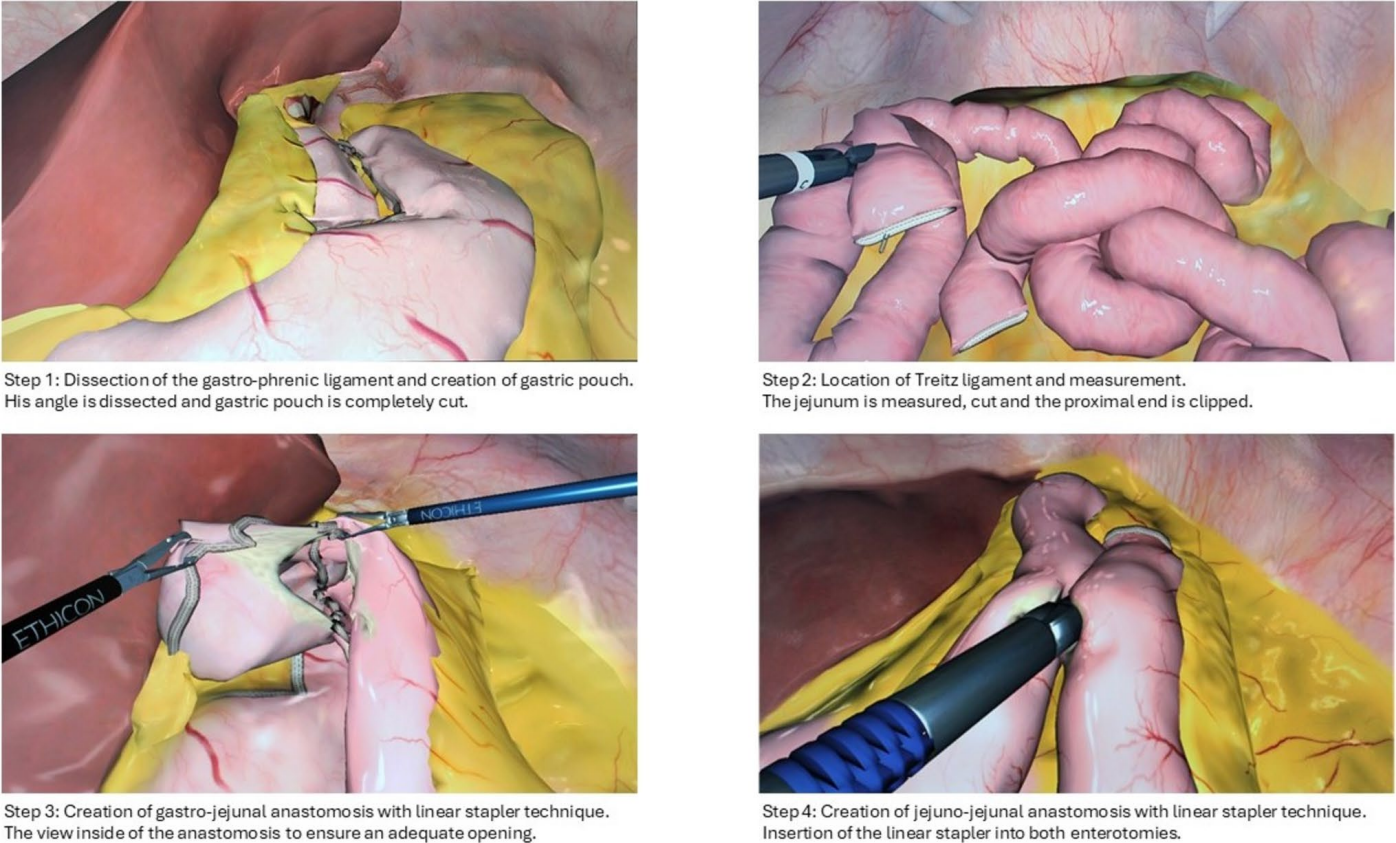
MIS RYGB steps on the VR trainer (Simbionix LAP Mentor)

The participants were asked to complete a questionnaire regarding their demographic data, experience in MIS, the time (years) passed since absolving the  basic MIS (MIS I) training module, and their notes in the basic MIS (MIS I) training module (Fig. 2).

### Training curriculum (a total of 4 h) for all participants:

1. **E-learning programs for basic laparoscopic training and MIS RYGB:** http://www.websurg.com [28]: Fully robotic Roux-en-Y gastric bypass | WebSurg, the online university of IRCAD.
2. The trainees were asked to study anatomy, illustrations, and videos of the MIS RYGB procedural techniques.
3. And to watch the “Laparoscopic RYGB” at https://youtu.be/Kpx_LKvl8Wo and https://youtu.be/p_-Rmk8Mfwk.
4. **Exercises on the VR Trainer** (detailed description provided in Supplementary Material 2):
5. Basic MIS skill module.
6. Four-step MIS RYGB module: Trained tutors instructed and trained in all individual steps of the MIS RYGB.

### Randomization and training

After completing the initial predefined training, the participants were randomly divided into the intervention and the control group using a free available randomization tool (https://ctrandomization.cancer.gov/tool/). The intervention group (n = 30) was split into fifteen pairs, while the participants in the control group (n = 30) trained solo.

**Fig. 3:**
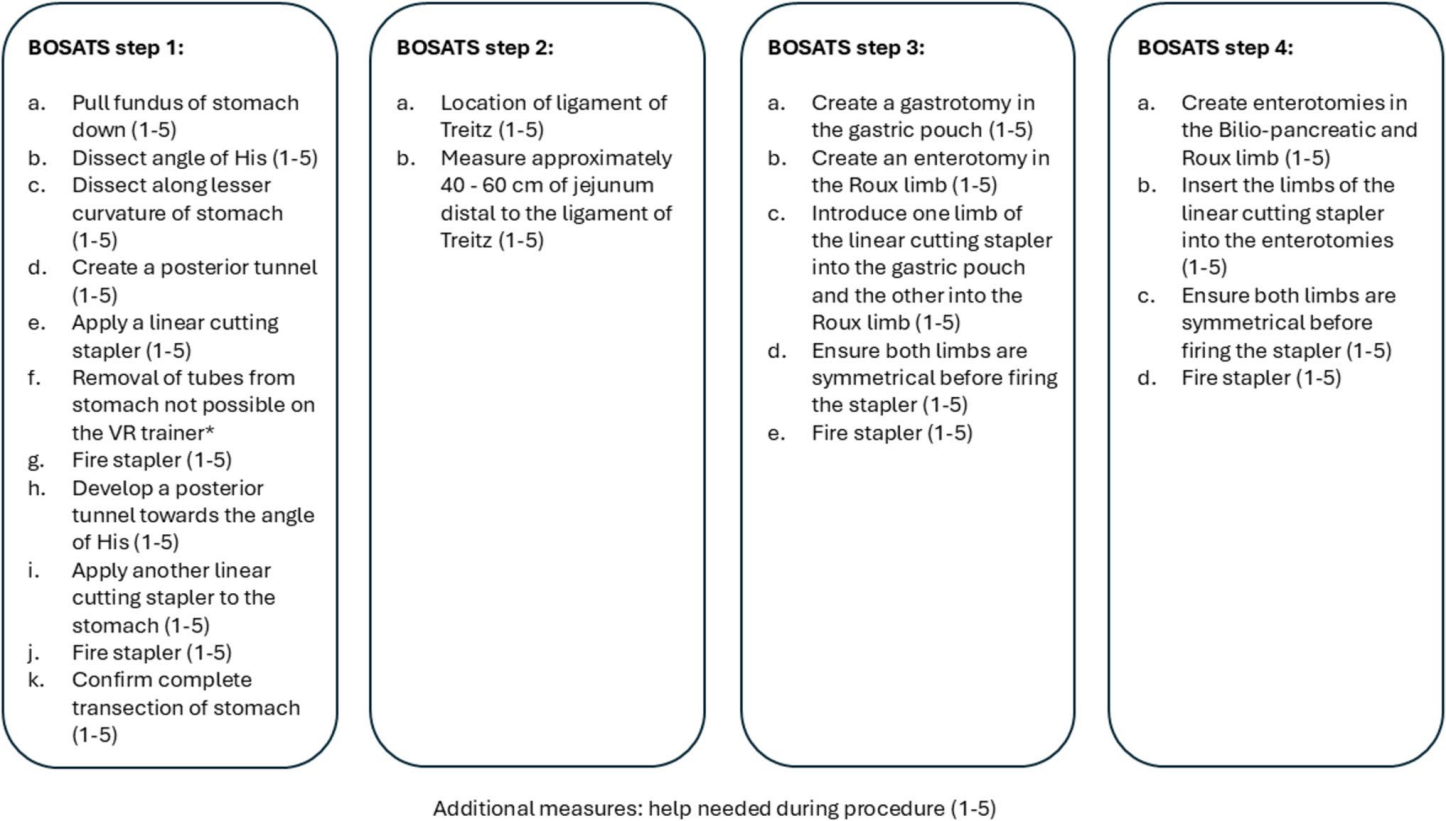
Description of the four MIS RYGB steps assessed by the BOSATS score. *The step was not included in the assessment since it cannot be performed on the VR trainer

Both groups trained MIS RYGB on a VR trainer and were assessed by the trained tutors using the BOSATS score. BOSATS score was developed to objectively evaluate the operative performance of MIS RYGB (Fig. 3) [29, 30].

The training was completed when the participants reached the proficiency level in MIS RYGB, which the trained tutors assessed. As there is no reported proficiency BOSATS score in the current literature, proficiency was defined as a BOSATS score ≥ 105 out of 110 for this study. However, if they did not get the necessary proficiency score evaluated by the trained tutors, they had to repeat the training. The re-entry of the training phase was repeated until the participants reached proficiency assessed by the trained tutors.

### Primary outcome

The study’s primary outcome was the difference between the total number (n) of training repetitions between the two groups needed to reach MIS RYGB proficiency according to the BOSATS score.

### Secondary outcomes

Secondary outcomes included comparison of BOSATS scores (for the whole surgical procedure and individual steps), comparison of performance time in minutes (total and per task), automatic assessments by VR Trainer (number of instrument movements, total instrument pathway in cm, and average instrument speed in cm/s), comparison of the number of bleeding complications (defined as n ≥ 2/procedure) determined by the VR trainer, and comparison of the validated score on current motivation (Questionnaire on Current Motivation or QCM) between the groups [31].

### Statistics

Statistical analysis and descriptive statistics were performed with the SPSS software (version 25.0, IBM SPSS Inc., Chicago, Illinois, USA), and data were given as absolute frequency and mean ± standard deviation. Differences between the data were assessed using the *t*-test for independent samples in parametric data and the Mann–Whitney U test for independent samples in the case of non-parametric data. For binary endpoints, group differences were calculated using the Chi-square test. A p-value of p < 0.05 was considered statistically significant. Visual representations were created using the Prism-GraphPad Software.

## Results

The study included sixty (n = 60) participants, who were randomly divided into the intervention (n = 30) and the control (n = 30) group. There were no differences in the demographic data between the groups (Table 1).

**Table 1:** Demographic parameters between the two groups

		Control Group (n = 30)	Intervention Group (n = 30)	P-value
Age (years)		24.2 ± 3.5	24.4 ± 4.7	0.828
Dominant hand	Right	28 (93.3%)	28 (93.3%)	1.000
Surgical experience		21 (70.0%)	18 (60.0%)	0.417
	Watching	11 (36.7%)	7 (23.3%)	0.260
	Assisting	9 (30.0%)	11 (36.7%)	0.584
	Performing	1 (3.3%)	0 (0.0%)	0.313
MIS experience		15 (50.0%)	11 (36.7%)	0.297
	Watching	7 (23.3%)	3 (10.0%)	0.166
	Assisting	7 (23.3%)	8 (26.7%)	0.766
	Performing	1 (3.3%)	0 (0.0%)	0.313
Playing videogames		6 (20.0%)	11 (36.7%)	0.152
Playing an instrument		9 (30.0%)	11 (36.7%)	0.584
MIS I completion*		0.7 ± 1.1	0.8 ± 1.1	0.905
MIS I grade	1	21 (70.0%)	18 (60.0%)	0.417
	2	9 (30.0%)	12 (40.0%)	0.417

*MIS* Minimally invasive surgery; ^*^years after completion. Data are presented as number (percentage) for categorical variables, mean standard deviation ± for normally distributed or median, and [25th and 75th percentile] for not normally distributed continuous variables. Accordingly, Chi-Quadrat, exact Fisher, Student’s t-test, or Mann–Whitney U test was used to compare

### Primary outcome

**Fig. 4:**
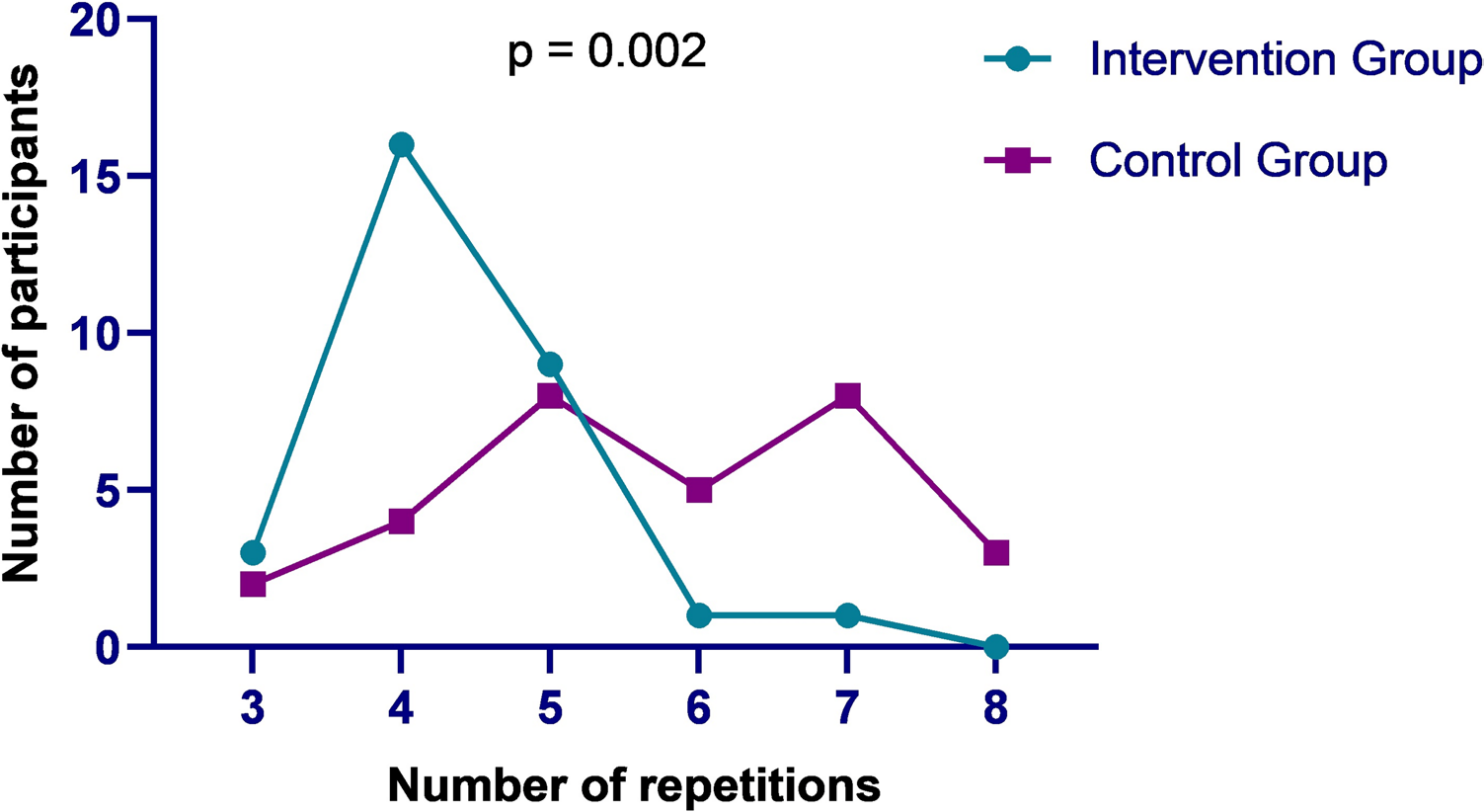
Comparison of the repetitions of MIS RYGB training needed until the two groups reached procedural proficiency

The intervention group needed significantly fewer repetitions to reach MIS RYGB proficiency than the control group (p = 0.002). Most participants in the intervention group reached proficiency after the fifth repetition, and none needed the eighth repetition. The control group reached proficiency much slower, with more participants needing sixth, seventh, and even eighth repetition than the participants of the intervention group (Fig. 4). Intention-To-Treat (ITT) analysis showed the intervention was most effective in the fourth repetition. The intervention completely lost its effect after the fifth repetition. The Per Protocol (PP) analysis showed that the intervention lost its impact after the sixth repetition (Table 2).

**Table 2:** Comparison of the ITT and Per Protocol analyses for different MIS RYGB repetitions

ITT			
Number of repetitions*	RD	RR	OR
4	0.40	4.1	7.3
5	0.07	1.3	1.4
6	− 0.17	0.15	0.13
7	− 0.24	0.11	0.09
8	− 0.10	Not Applicable**	Not Applicable**
Per protocol***			
4	0.45	4.2	8.7
5	0.52	2.8	10.9
6	0.15	1.4	1.8
7	0.28	1.4	0.4
8	Not Applicable****	Not Applicable****	Not Applicable****

*ITT*, intention-to-treat effect; *MIS RYGB* minimally invasive Roux-en-Y-Gastric Bypass; ^*^to reach proficiency; *RD*, risk difference; *RR*, risk ratio; *OR*, odds ratio; **undefined since success in the intervention group is 0; ***excluding participants that reached proficiency (“not receiving intervention”); ****not calculated since success in the control group is 100% and in the intervention group 0%

### Secondary outcomes

The initial BOSATS scores were comparable between the two groups (Table 3). However, the intervention group performed better than the control group in the second, fourth, and fifth MIS RYGB (91.1 ± 6.4 vs. 87.1 ± 7.0 points, p = 0.025; 104.0 ± 4.7 vs. 100.3 ± 6.1 points, p = 0.014; 106.2 ± 2.8 vs. 101.9 ± 5.8 points, p = 0.026), respectively.

**Table 3:** Comparison of the BOSATS scores between the two groups

BOSATS score	Control Group (n = 30)	Missing Values*	Intervention Group (n = 30)	Missing Values*	P-value
*First RYGB*	74.3 ± 6.3	0	75.8 ± 7.1	0	0.959
Step 1	33.8 ± 4.0		33.6 ± 3.5		0.838
Step 2	6.7 ± 1.2		7.1 ± 1.2		0.244
Step 3	16.2 ± 2.1		16.4 ± 2.8		0.756
Step 4	15.8 ± 1.9		15.9 ± 1.9		0.840
*Second RYGB*	87.1 ± 7.0	0	91.1 ± 6.4	0	**0.025**
Step 1	39.3 ± 4.1		40.9 ± 2.7		0.068
Step 2	8.3 ± 1.2		8.6 ± 1.0		0.210
Step 3	19.1 ± 2.5		19.7 ± 2.7		0.346
Step 4	16.7 ± 1.7		17.5 ± 1.5		0.058
*Third RYGB*	96.1 ± 7.4	0	98.3 ± 6.2	0	0.226
Step 1	43.5 ± 3.7		44.7 ± 3.4		0.194
Step 2	8.9 ± 1.3		9.4 ± 0.9		0.108
Step 3	20.9 ± 2.5		21.2 ± 2.2		0.588
Step 4	18.2 ± 1.5		18.2 ± 1.6		0.935
*Fourth RYGB*	100.3 ± 6.1	2	104.0 ± 4.7	3	**0.014**
Step 1	45.4 ± 2.7		45.6 ± 9.2		0.875
Step 2	9.4 ± 0.8		9.8 ± 0.5		0.068
Step 3	22.2 ± 1.9		23.1 ± 1.4		0.057
Step 4	18.6 ± 1.6		19.0 ± 1.1		0.291
*Fifth RYGB*	101.9 ± 5.8	6	106.2 ± 2.8	19	**0.026**
Step 1	45.8 ± 3.8		48.3 ± 1.5		**0.041**
Step 2	9.5 ± 1.0		9.9 ± 0.3		0.253
Step 3	22.9 ± 1.9		23.5 ± 1.1		0.282
Step 4	18.8 ± 1.1		19.6 ± 0.5		**0.048**
*Sixth RYGB*	104.3 ± 4.2	13	106.5 ± 2.1	28	0.480
Step 1	47.2 ± 2.6		48.0 ± 0.0		0.668
Step 2	9.9 ± 0.3		10.0 ± 0.0		0.631
Step 3	23.3 ± 1.1		24.0 ± 1.4		0.413
Step 4	19.2 ± 1.2		19.5 ± 0.7		0.767

*BOSATS*, Bariatric Objective Structured Assessment of Technical Skills; ^*^due to reaching proficiency; *RYGB*, Roux-en-Y-Gastric Bypass. Data are presented as number (percentage) for categorical variables, mean standard deviation ± for normally distributed or median, and [25th and 75th percentile] for not normally distributed continuous variables. Accordingly, Chi-Quadrat, exact Fisher, Student’s t-test, or Mann–Whitney U test was used to compare

P values < 0.05 are marked in bold

The intervention group had fewer bleeding incidents in the fifth and sixth MIS RYGB repetition than the control group (2 vs. 10, p = 0.001; 0 vs. 8, p < 0.001, respectively) (Table 4).

**Table 4:** Comparison of the bleeding incidents between the two groups

	Control Group (n = 30)	Missing Values*	Intervention Group (n = 30)	Missing Values*	P-value
Bleeding incidents**					
First RYGB	20 (66.7%)	0	20 (66.7%)	0	1.000
Second RYGB	16 (53.3%)	0	17 (56.7%)	0	1.000
Third RYGB	13 (43.3%)	0	13 (43.3%)	0	1.000
Fourth RYGB	13 (46.4%)	2	7 (25.9%))	3	0.257
Fifth RYGB	10 (41.7%)	6	2 (18.1%)	19	**0.001**
Sixth RYGB	8 (47.1%)	13	0 (0.0%)	28	** < 0.001**

^*^Due to reaching proficiency; **Defined as the number (n) of bleeding incidents of n ≥ 2/procedure; *RYGB*, Roux-en-Y-Gastric Bypass. Data are presented as number (percentage) for categorical variables, mean standard deviation ± for normally distributed or median, and [25 th and 75 th percentile] for not normally distributed continuous variables. Accordingly, Chi-Quadrat, exact Fisher, Student’s t-test, or Mann–Whitney U test was used to compare

P values < 0.05 are marked in bold

There was no difference in performance time between the two groups (Table 5). Attempts 7 and 8 were not calculated because they missed the majority (attempt 7) or completely (attempt 8) of the intervention group. Additionally, the difference between the number of instrument movements and the total instrument pathway in centimeters favored the intervention group only during the observation of the fourth MIS RYGB (Table 5).

**Table 5:** Comparison of the performance time and VR scores between the two groups

	Control Group (n = 30)	Missing Values	Intervention Group (n = 30)	Missing Values	P -value
*Time (min:s)*					
First RYGB	36:23 ± 06:34	0	38:25 ± 11:30	0	0.407
Second RYGB	25:55 ± 06:57	0	26:28 ± 05:34	0	0.738
Third RYGB	21:57 ± 05:41	0	21:58 ± 06:16	0	0.988
Fourth RYGB	19:38 ± 04:36	2	19:10 ± 04.49	3	0.714
Fifth RYGB	19:12 ± 05:19	6	18:56 ± 03:59	19	0.877
Sixth RYGB	19:24 ± 05:13	13	18:29 ± 07:51	28	0.823
*Number of instrument movements*					
First RYGB	2357.7 ± 555.3	0	2428.2 ± 725.6	0	0.674
Second RYGB	1787.0 ± 534.7	0	1709.0 ± 445.6	0	0.542
Third RYGB	1522.8 ± 465.9	0	1414.1 ± 428.2	0	0.351
Fourth RYGB	1356.9 ± 374.2	2	1168.9 ± 247.7	3	**0.033**
Fifth RYGB	1334.1 ± 456.9	6	1127.0 ± 397.8	19	0.205
Sixth RYGB	1312.3 ± 483.8	13	1293.5 ± 647.0	28	0.960
*Total instrument pathway (cm)*					
First RYGB	4188.2 ± 1266.3	0	4280.5 ± 1448.3	0	0.794
Second RYGB	3268.5 ± 1186.9	0	3108.8 ± 956.0	0	0.568
Third RYGB	2846.4 ± 849.9	0	2515.0 ± 841.4	0	0.135
Fourth RYGB	2551.0 ± 655.0	2	2239.6 ± 3419.5	3	**0.041**
Fifth RYGB	2495.0 ± 726.7	6	2051.5 ± 541.8	19	0.081
Sixth RYGB	2535.1 ± 889.0	13	2168.6 ± 833.7	28	0.587
*Average instrument speed (cm/s)*					
First RYGB	20.4 ± 0.7	0	20.2 ± 0.5	0	0.186
Second RYGB	20.7 ± 0.7	0	20.5 ± 0.6	0	0.287
Third RYGB	20.8 ± 0.8	0	20.5 ± 0.6	0	0.158
Fourth RYGB	21.0 ± 0.7	2	20.8 ± 0.7	3	0.533
Fifth RYGB	21.0 ± 0.7	6	20.6 ± 0.6	19	0.074
Sixth RYGB	21.1 ± 0.8	13	20.8 ± 0.6	28	0.592

*VR*, Virtual reality; *RYGB*, Roux-en-Y-Gastric Bypass. Data are presented as number (percentage) for categorical variables, mean standard deviation ± for normally distributed or median, and [25 th and 75 th percentile] for not normally distributed continuous variables. Accordingly, Chi-Quadrat, exact Fisher, Student’s t-test, or Mann–Whitney U test was used to compare

P values < 0.05 are marked in bold

**Fig. 5:**
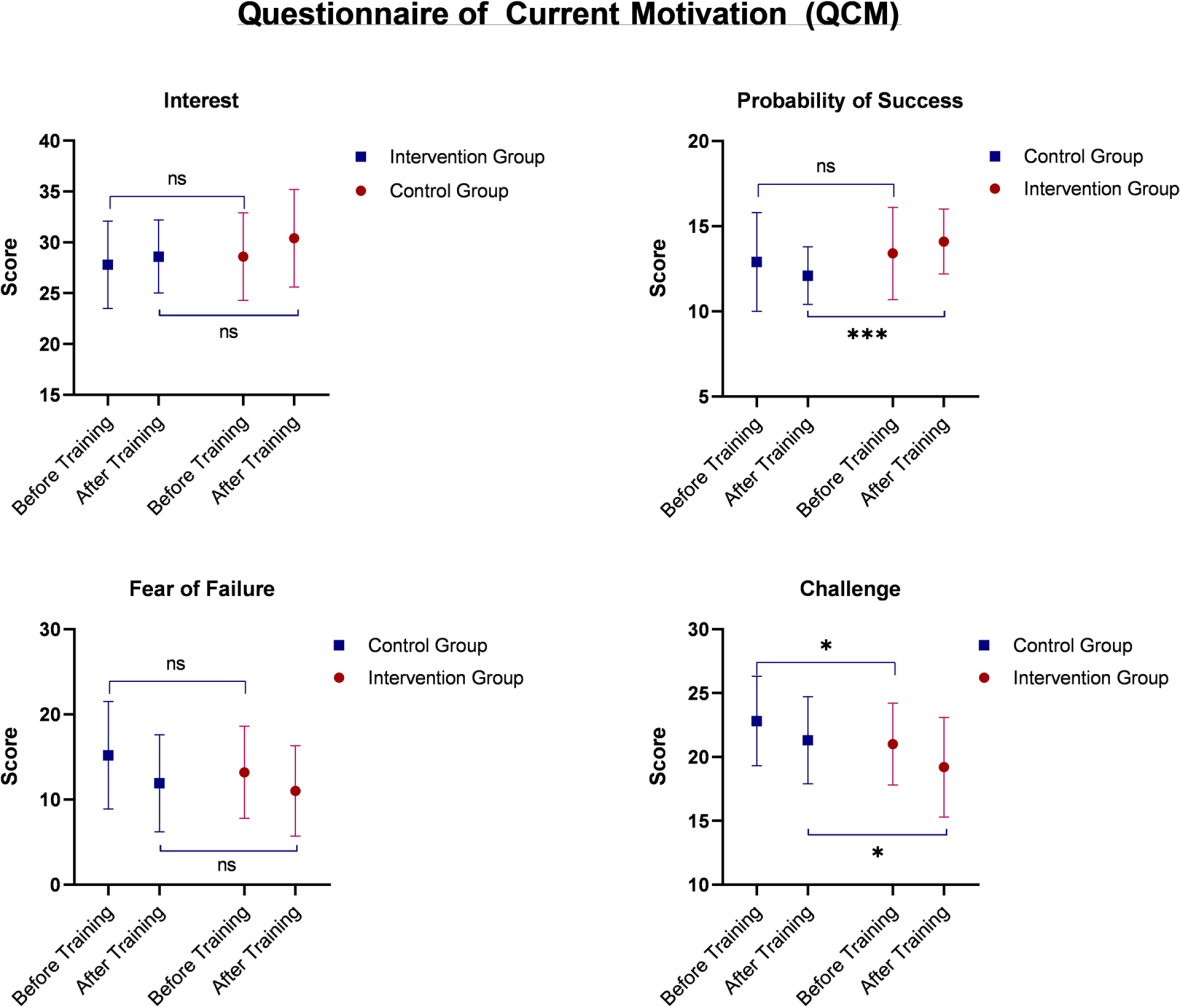
Comparison of the Questionnaire on Current Motivation (QCM) between the two groups before and after the completion of the training

After completing their training, the participants in the intervention group were more confident of their successful MIS RYGB performance than the participants in the control group (Fig. 5). However, they saw the procedure as less challenging before and after the training than the control group. There were no differences in the procedure’s interest or the participants’ fear of failure before and after training between the two groups (Fig. 5).

When comparing the individual participants from both groups, the participants from the intervention group performing MIS RYGB after their partner had higher BOSATS scores after the first MIS RYGB compared to their partners (78.5 ± 7.0 vs. 73.1 ± 6.3, p = 0.032). These participants outperformed the participants in the solo group in the fourth MIS RYGB (104.4 ± 4.5 vs. 100.3 ± 6.1, p = 0.031). There were no further differences in BOSATS scores (Table 6).

**Table 6:** Comparison of BOSATS scores between individual participants from both groups

	Solo group (n = 30)	Missing values	First participant* (n = 15)	Missing values	Second participant* (n = 15)	Missing values
BOSATS Score						
First RYGB	74.8 ± 6.4	0	73.1 ± 6.3	0	78.5 ± 7.0	0
Second RYGB	87.1 ± 7.0	0	91.0 ± 7.0	0	91.3 ± 6.0	0
Third RYGB	96.1 ± 7.4	0	97.5 ± 5.4	0	98.9 ± 7.1	0
Fourth RYGB	100.3 ± 6.1	2	103.5 ± 5.1	2	104.4 ± 4.5	1
Fifth RYGB	101.9 ± 5.8	6	106.5 ± 1.4	9	105.8 ± 4.1	10
Sixth RYGB	104.3 ± 4.2	13	/	15	106.5 ± 2.1	13

	P-value Solo vs. First	P-value Second vs. Solo	P-value Second vs. First
BOSATS Score			
First RYGB	0.403	0.079	**0.032**
Second RYGB	0.087	0.053	0.890
Third RYGB	0.500	0.222	0.546
Fourth RYGB	0.104	**0.031**	0.633
Fifth RYGB	0.661	0.164	0.701
Sixth RYGB	/	0.480	/

*BOSATS*, Bariatric Objective Structured Assessment of Technical Skills; *RYGB*, Roux-en-Y-Gastric Bypass; /, not calculated due to reaching proficiency. Data are presented as number (percentage) for categorical variables, mean standard deviation ± for normally distributed or median, and [25th and 75th percentile] for not normally distributed continuous variables. Accordingly, Chi-Quadrat, exact Fisher, Student’s t-test, or Mann–Whitney U test was used to compare

*P* values < 0.05 are marked in bold

## Discussion

The present randomized-controlled study showed that training MIS RYGB on a VR trainer in pairs contributed to achieving procedural proficiency with fewer repetitions than training alone. Furthermore, the intervention group had fewer complications and better performance scores than the control group.

Optimizing MIS training is essential because it requires a particular skill set compared to open surgery [32]. Simulation training in MIS, such as training on a VR trainer, has proven to be a safe and efficient method of learning MIS [17, 21, 33, 34]. This part of MIS training is precious when it comes to more complex MIS procedures, such as MIS RYGB, because it enables surgical novices to learn and advance their skills in a safe environment [35–38].

Additionally, optimizing the workspace in MIS training to be more efficient has become a priority for many educational researchers [39–42]. Kowalewski et al. demonstrated the benefits of training in pairs in a multimodality MIS training curriculum [25]. The group that trained in pairs performed ex-vivo porcine laparoscopic cholecystectomy faster than the participants who trained solo. Moreover, the intervention group had fewer exercise repetitions on the VR trainer compared to the control group. These findings correspond to the results of the presented study since the intervention group needed fewer exercise repetitions before reaching MIS RYGB proficiency compared to the control group. However, Kowalewski et al. included MIS novices, in contrast to the present research, which included participants who had acquired basic MIS skills in a predefined structured MIC module. Hence, the reported results could be replicated in surgical residents and improve their MIS training. This could further lead to a reduced learning curve in MIS RYGB in a clinical setting and improved patient outcomes. However, further studies need to examine this hypothesis and include surgical residents as participants to test the validity of the reported results.

Furthermore, it is important to note that while the PP analysis overestimated the effects of the intervention by indicating that the effects remain noticeable at the sixth repetition, the ITT analysis strongly emphasizes the positive impact of the intervention after the fourth repetition, and somewhat less after the fifth repetition. This confirms the positive effects on achieving MIS RYGB proficiency after the fourth and fifth repetitions compared to the control group. The automatic assessment of the number of instrument movements and of the total instrument pathway supports this conclusion, underlying the benefit of the intervention in reaching proficiency after the fourth MIS RYGB.

Not only did the intervention group need fewer exercise repetitions until reaching proficiency, but it also performed better on several MIS RYGB rounds compared to the control group. Improvement not only in the time needed to reach proficiency but also in procedural performance itself advocates for the undeniable benefit of training in pairs when it comes to MIS RYGB on a VR trainer. Witnessing the procedure from an assistant surgeon’s safe and more passive perspective and then transferring that knowledge when taking over the leading surgeon’s position could be the cause of performance improvement. The trainees might have felt more confident performing the procedure and retaining the knowledge longer simply through the exposure to the procedure and through communication with their partner during training [43–46]. Bjerrum et al. reported that training in pairs was as effective as training solo in learning bronchoscopy while requiring the same resources and training time [44]. These results imply that training in pairs can improve the cost-effectiveness of MIS RYGB simulation training on a VR trainer since it provides superior results compared to solo training. It is reasonable to argue that training in pairs should be included in the training curricula, particularly in simulation MIS training, because of its cost-effectiveness, lower cognitive load, and benefits for performance improvement.

Analyzing the complication rates during the MIS RYGB repetitions, the intervention group had fewer bleeding incidents in later repetitions than the control group. The reduction in bleeding incidents in the intervention group could be due to the observation of the errors of the training partner and learning how to avoid them when performing the procedure. The benefits of training in pairs were reported not only by observing peers while they train, but also through technical discussions between the training partners, analyzing errors, and knowledge exchange [47–49]. Ritchie et al. reported that training laparoscopic skills in pairs led to increased peer support, feedback, and observation time compared to individual training [50]. All these elements could have contributed to the superior results of the intervention group compared to the control group. However, it is essential to mention that these benefits are still limited to the simulation training and might not be transferred to the clinical setting since the training partners are often not peers but rather a combination of an experienced surgical mentor and a surgical trainee. This can hinder the benefits of learning from errors and discussions between peers, often present in the training in pairs [51].

Lastly, the intervention group was more confident of their performance by the end of their training than the control group. Kirchner et al. reported that training in pairs can reduce the cognitive load since the cognitive load is shared between the training partners, enhancing the learning process [52]. Räder et al. described that overt communication and cooperation between partners while training in pairs to learn coronary angiography contributed to their learning, underlining the importance of peer feedback and interaction when learning a new skill [51]. These cognitive aspects of training in pairs are essential to building confidence while acquiring new surgical skills and improving learning effects.

### Limitations

Although the study included medical students with previous MIS knowledge obtained through a predefined multimodal basic MIS course, the question remains whether these results are transferable to surgical residents. Surgical residents with experience in the operating room might have reached MIS RYGB proficiency alone for the same amount of time as their peers in pairs. However, using medical students instead of residents can be considered a study’s strength rather than a limitation. If training in pairs enhances training, then this should be incorporated earlier in medical education to make training more efficient and scalable, especially in the global surgery setting. Further clinical research should test the benefits of training in pairs in a clinical setting with surgical residents.

Additionally, the training of MIS RYGB on a VR trainer could influence the reproducibility of the results due to the safe and controlled practice environment, which is often not replicable in the actual clinical setting. While VR trainers provide convenient access and automated assessment of surgical skills, their visual and haptic feedback remains constrained compared to alternative training modalities, such as animal and cadaver models.

However, simulation training is essential in learning any MIS procedures since it provides an opportunity to practice and advance MIS skills before performing a procedure in the operating room. Therefore, simulation training should always precede performing MIS procedures in the operating room due to its learning benefits and confidence boost. A further limitation of the study is the establishment of a BOSATS proficiency score set at 105 points. This particular proficiency score has not undergone validation, which could have affected the study’s outcomes. Nonetheless, there is currently no existing evidence in the literature regarding the BOSATS proficiency score, necessitating its definition for the purposes of this study. A potential observer bias exists because the tutors conducting the study were not blinded. Nevertheless, this issue could not be avoided due to their roles in training the participants.

## Conclusion

Training MIS RYGB in pairs on a VR trainer enabled trainees to reach procedural proficiency with fewer exercise repetitions than training alone in a sample of medical students. Furthermore, training in pairs reduced complication rates and improved performance according to the BOSATS score. Clinical transfer should be assessed since training in pairs in a clinical setting could potentially reduce the learning curve not only for simple MIS skills but also for more complex MIS procedures such as MIS RYGB.

## Supplementary Information

Below is the link to the electronic supplementary material.

Supplementary file1 (DOCX 1512 kb)
