# Supplementary material for: Effects of training in pairs versus training alone on reaching proficiency in minimally invasive Roux-en-Y-gastric bypass on a virtual reality trainer in medical students: a randomized-controlled trial
Source: Surg Endosc. 2025 Apr 14;39(5):3285–97. doi: 10.1007/s00464-025-11701-9 (PMC12041044; doi:10.1007/s00464-025-11701-9)

**Training curriculum**

**Basic skills task:**

- To reactivate their VR trainer knowledge, students were asked to perform a small introductory exercise
- Students were instructed to grasp a flexible circle and pull it upwards to expose the strings attached to it
- The strings must be cut with scissors to free the circle completely
- Grasping the strings with different hands/instruments in particular order to practice dexterity

**
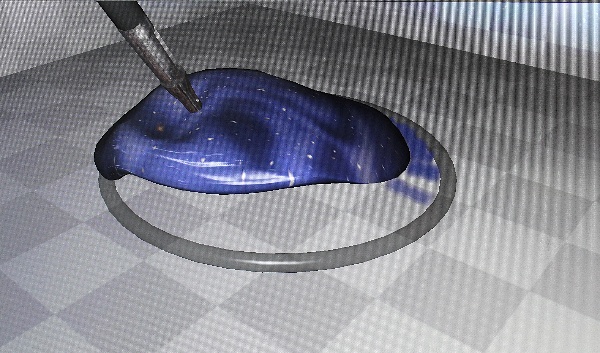

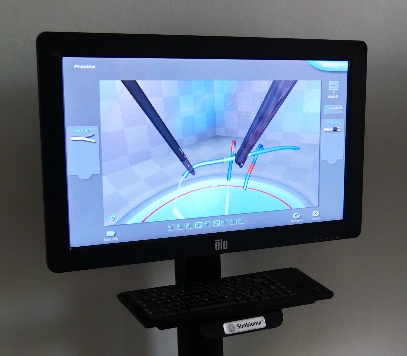
**

**Figure 1:** Grasping a flexible circle (on the left) and Grasping the strings in particular order with different hands/instruments

**Roux-Y-Gastric Bypass:**

1. Step :

- Get an overview of the surgical field and set the camera
- Find and dissect the angle of His
- Move along the small curvature, dissect between the second and third gastric artery and create a posterior tunnel
- Insert the small linear cutting stapler horizontally
- Confirm the right angle and fire the linear cutting stapler
- Create another posterior tunnel towards the angle of His
- Insert the bigger linear cutting stapler vertically
- Confirm the right angle and fire the linear cutting stapler
- Confirm the complete transection of the stomach

1. Step:

- Locate the ligament of Treitz
- Measure approximately 40 to 60 cm of jejunum distal to the ligament of Treitz
- Introduce the linear cutting stapler and cut through the jejunum
- Apply a clip to the proximal end of the jejunum
- Grasp the distal end of the jejunum and measure an additional 100 cm from there

1. Step:

- Create a gastrotomy in the gastric pouch
- Create an enterotomy in the Roux limb
- Insert one leg of the linear cutting stapler into the gastrotomy and the other into the enterotomy
- Ensure symmetry and fire the linear cutting stapler

1. Step:

- Create an enterotomy in the bilio-pancreatic and in the Roux limb
- Insert one leg of the linear cutting stapler in each enterotomy
- Ensure symmetry and fire the linear cutting stapler


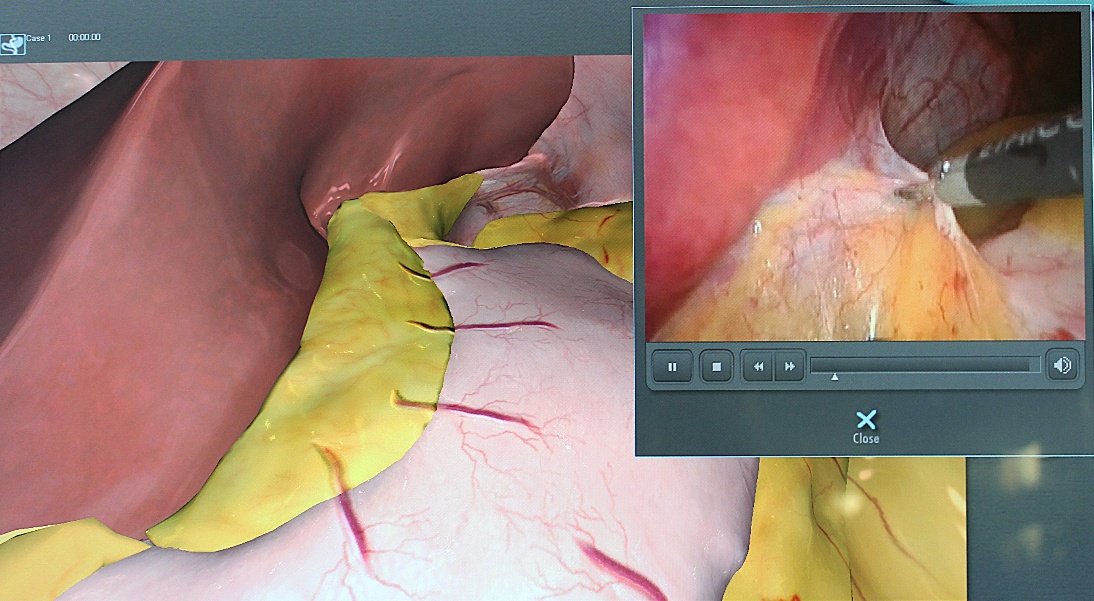

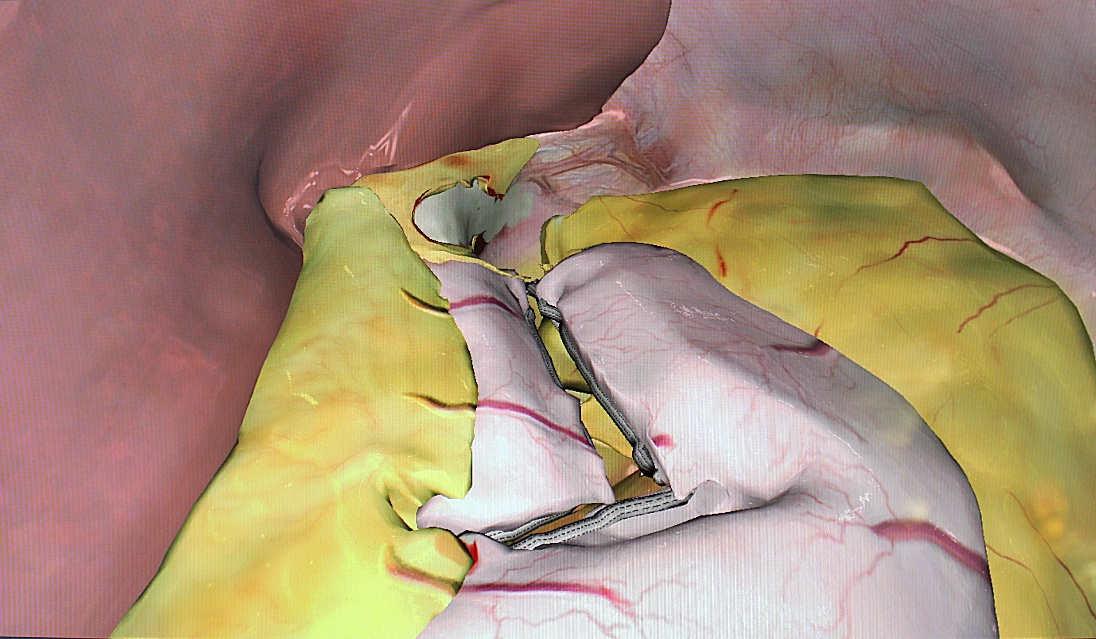

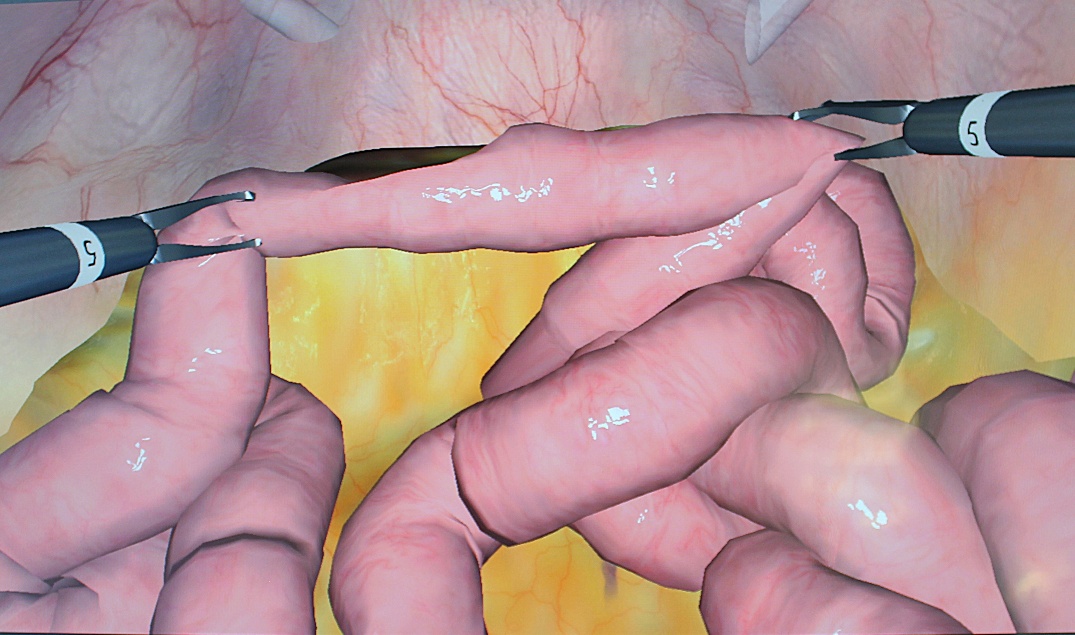

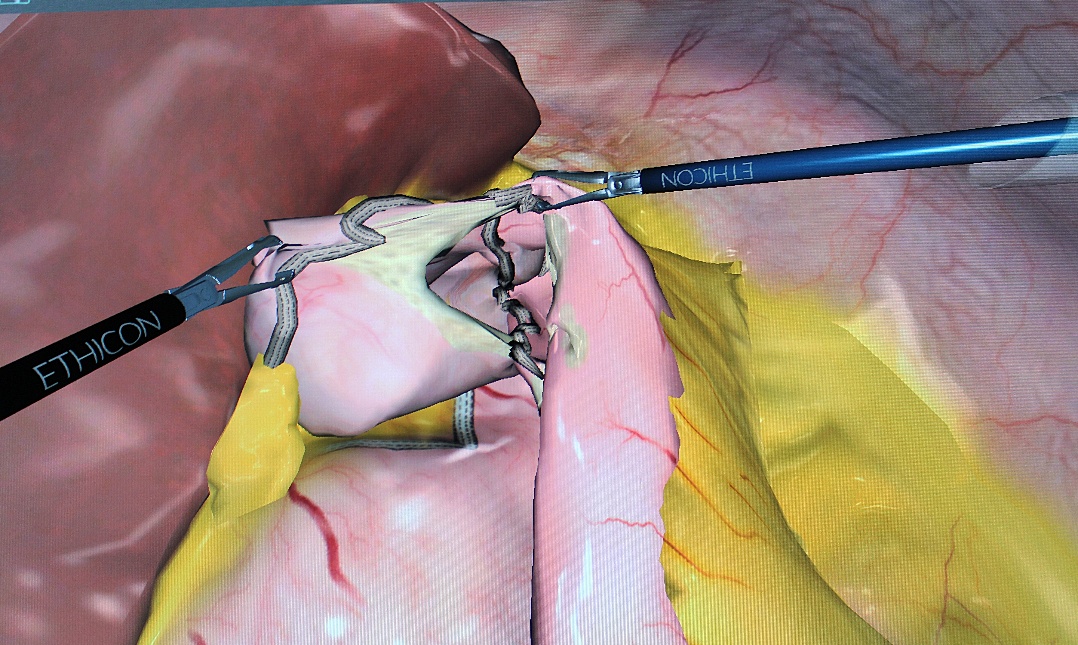

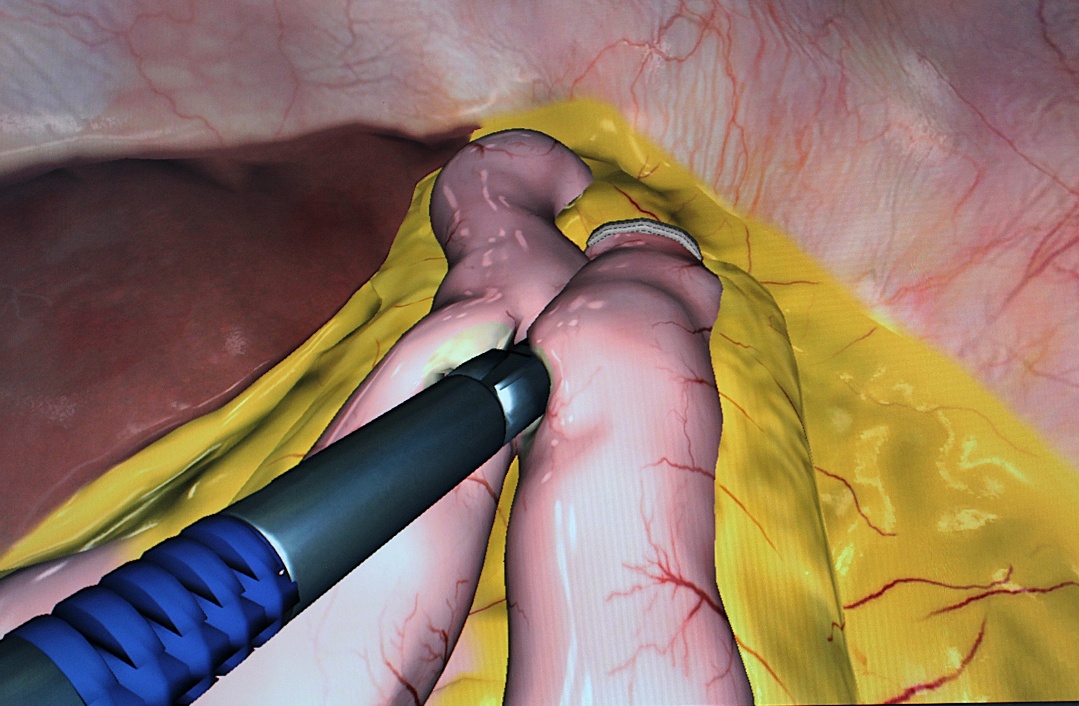

Supplement: Supplementary file 1 — Supplementary file1 (DOCX 1512 kb) [file 464_2025_11701_MOESM1_ESM.docx]
